# Optimized Degradation of Eosin Dye Through UV-ZnO NPs Catalyzed Reaction

**DOI:** 10.1007/s10895-022-02889-3

**Published:** 2022-01-19

**Authors:** Rania Farouq, Ehsan Kh. Ismaeel, Aliaa M. Monazie

**Affiliations:** 1 Petrochemical Engineering Department, Pharos University in Alexandria, Alexandria, Egypt; 2 El Minia High Institute of Engineering and Technology, El Minia, Egypt; 3 Faculty of Engineering, Fayoum University, Fayoum, Egypt

**Keywords:** Eosin dye, Photocatalyst, Treatment, Nanoparticle, Wastewater

## Abstract

The present study is set out to determine the photocatalytic degradation potential of ZnO nanoparticles for effective degradation of Eosin dye. The heterogeneous photocatalytic experiments were carried out by irradiating aqueous dye solutions with ultraviolet light. The influence of effective parameters like flow rate, pH, catalyst dose, and dye concentration was examined. The best degradation efficiency (66.82%) of ZnO Nanoparticles against Eosin dye was achieved within 90 min of reaction time. The Box–Behnken design under the Response Surface Methodology (RSM) was chosen as a statistical tool to obtain the correlation of influential parameters. The optimum values were recorded as follows: 0.59 g, 15.75 ppm and 136.12 ml/min for amount of catalyst, dye concentration and flow rate, respectively. The maximum percent degradation achieved at these conditions was 71.44%.

## Introduction

Water is extremely important for the continuation of life. Therefore, every decision and every step to be taken regarding water is vital [1]. Most of the pollutants in wastewater from industrial sources contain some organic chemicals and pathogens that must be removed or treated before being discharged into different water sources [2].

Synthetic dyes or colorings are basic chemicals for most industries that produce textiles, food, cosmetics, etc.… [3, 4]. As a result a huge amount of dyestuff-contaminated water is left from the above industries and dye color is the main recognized pollutant in wastewater [5].

The presence of very small amounts of dyes in water is very pronounced and affects the aesthetic merit, water transparency and gas solubility in various water bodies. Water decolourisation is often more important than the removal of colorless, soluble organic matter, which typically contributes to the bulk of the biochemical oxygen demand (BOD) [6, 7].

Eosin Y (or more commonly, eosin) is one of the dyes that pollutes water. Eosin is a water-soluble dye that is a red crystalline powder, frequently used in textile dyeing and ink manufacturing. It is an acidic pigment belonging to the xanthine group, which has a yellowish-red color with a greenish fluorescence [8].

Organic dyes are considered hazardous water pollutants, so there are several ways to remove them from the environment, which mainly depend on biological, chemical and physical methods [9].

Most of the conventional chemical, physical and biological processes used to treat water contaminated with textile dye have drawbacks such as high cost, high energy requirements, etc.… [10]. Therefore, it is urgent to find new and more efficient methods for treating wastewater contaminated with dyes.

In recent years, alternative technologies for treating dyes in industrial waters have been developed. These techniques are known as advanced oxidation processes (AOPs) [11, 12].

AOPs technologies have received wide attention for the decomposition of organic dyes. These technologies are based on the photo enhanced generation of highly reactive hydroxyl radicals, which oxidize the organic matter in solution and completely convert it to water, carbon dioxide and inorganic compounds [13]. Photocatalytic degradation, an AOP process that uses semiconductors such as titanium dioxide and zinc oxide to decompose organic pollutants, has been used in recent years as an effective alternative to treating dye-contaminated wastewater [14]. One of the unique features of the method of using semiconductors as a photocatalyst to remove pigments from polluted water is the complete mineralization into environmentally friendly products, without generating side waste [15]. Other advantages of semiconductors include ease of regeneration, reuse, and activity under readily available UV visible light. Zinc oxide is one of the best photocatalysts used recently [16].

Optimizing the quantities of the photocatalytic reagent plays a major role towards the success of the photocatalytic process [4, 17, 18]. The traditional method involves changing one variable (say the amount of Zn^2+^) while keeping the rest of the other variable influences constant, and studying the effect of this single variable on the response. This is very time consuming, expensive and complex for a multivariate system. To avoid these difficulties, a statistically based technique, called the response surface method (RSM) is used as a powerful experimental design tool that optimizes the factors influencing the photocatalytic process very precisely [19–22].

The present study is an investigation for the treatment of Eosin yellow dye wastewater by ZnO nano-particles as photocatalyst and for the parameters that affect the process. Also, optimization using RSM is conducted based on Box–Behnken design method.

## Experimental Work

### Experimental Setup

**Fig. 1:**
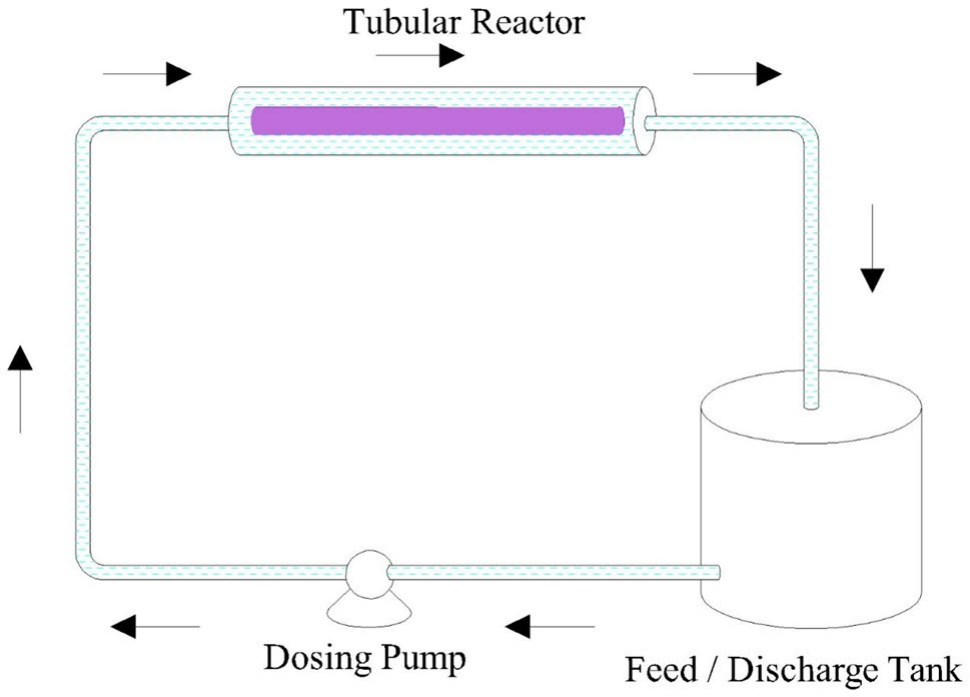
A schematic diagram for the experimental set-up

The experiments were carried out in a UV Tubular reactor, which photodegrades the EY dye using a UV lamp (Fig. 1). It consists of a feed-discharge container, pump and a tubular reactor. The reactor is 100 mm in diameter and 400 mm in length and it encloses a UV lamp of wavelength 400 nm and 15 watts

## Material and Method

### Material

#### Wastewater

**Fig. 2:**
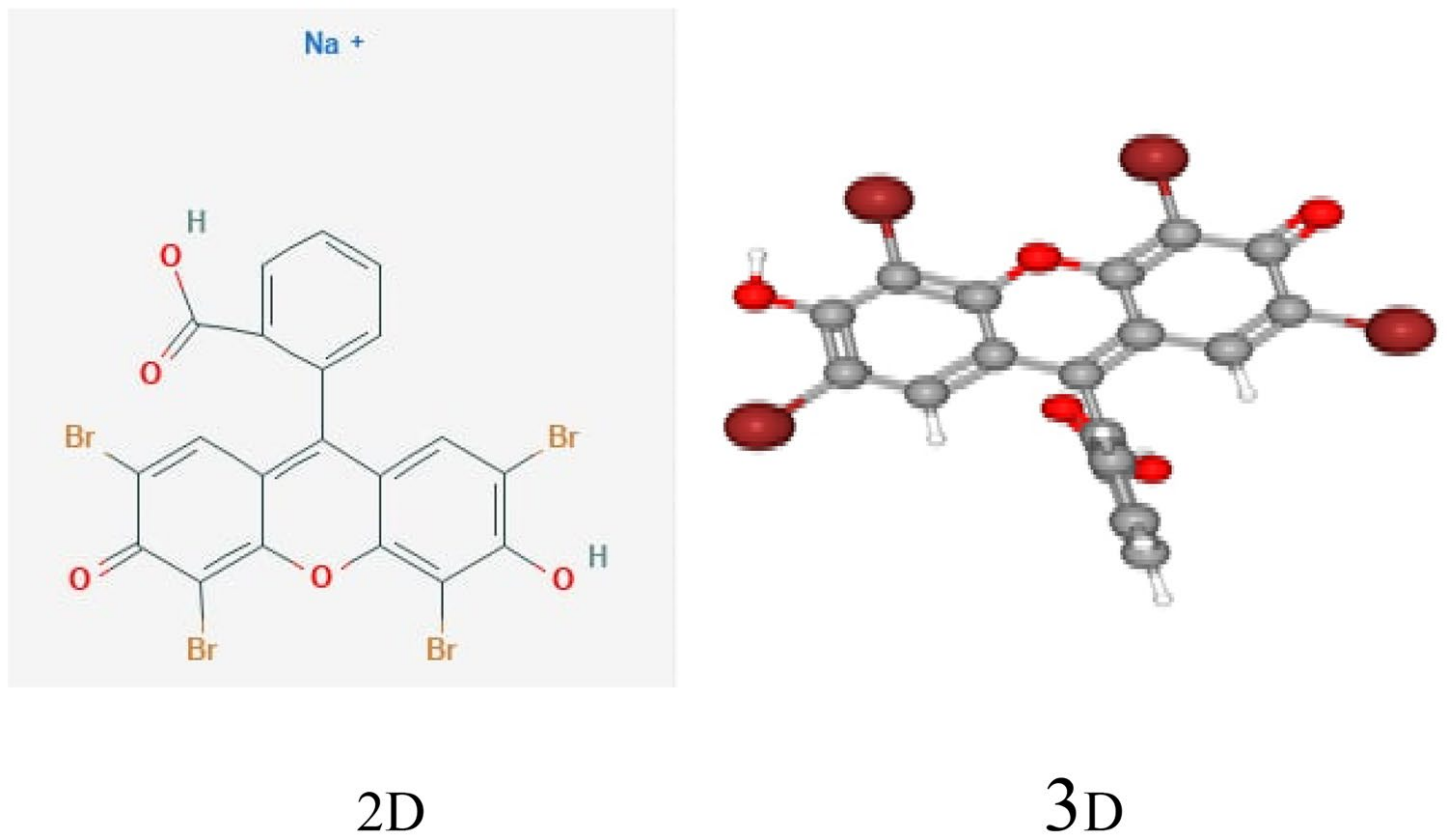
Structure of Eosin yellow dye

Eosin yellow dye (λ=517nm); which is routinely used in industry, is utilized to create a simulated dyestuff effluent. The structure of the dye is shown in Fig. 2.

#### Nano-Structured Photocatalyst

ZnO Nano particles with particle size <100nm and average particle size < 40nm are used as catalysts for the degradation of the pollutant, Eosin yellow dye; in the synthetic wastewater. The catalyst was prepared by Beni-Suef University.

#### Method

The contaminated water is circulated using only a pump (a Cole Parmer Instrument Company System 7521- 10 peristaltic (dosing) pump). A magnetic stirrer is employed to ensure that the catalyst is thoroughly mixed with the contaminated water. HCl and NaOH of analytical grade are used to adjust the initial pH which is measured by a digital pH meter (model type pH-2005). After specified periods of time, samples are withdrawn and centrifuged (centrifuge model type TDL-16B) to separate the catalyst from the solution before being measured for dye concentration using a spectrophotometer (PG Instruments LTD, Model T-80).

#### Experimental Design and Data Analysis

Response Surface Methodology based on Box-Behnken experimental design was applied for optimizing the process response (% Degradation), and to evaluate the effect of the independent process variables (Amount of catalyst, 0.2–0.6 g; Dye concentration, 15–85 ppm, Flow rate, 40–148 ml/min). Experimental process variables and levels for Box-Behnken design are given in Table 1. Design-Expert 10.0.1 software was used for the statistical analysis, modeling and optimization.

**Table 1:** Experimental variables and levels for Box-Behnken design

Variables	Symbols	Level		
		– 1	0	+ 1
Amount of catalyst (g)	A	0.2	0.4	0.6
Dye concentration (ppm)	B	15	50	85
Flow rate (ml/min)	C	40	94	148

## Results and Discussion

### Effect of UV Irradiation in the Absence of Catalyst

#### Effect of Flow Rate

**Fig. 3:**
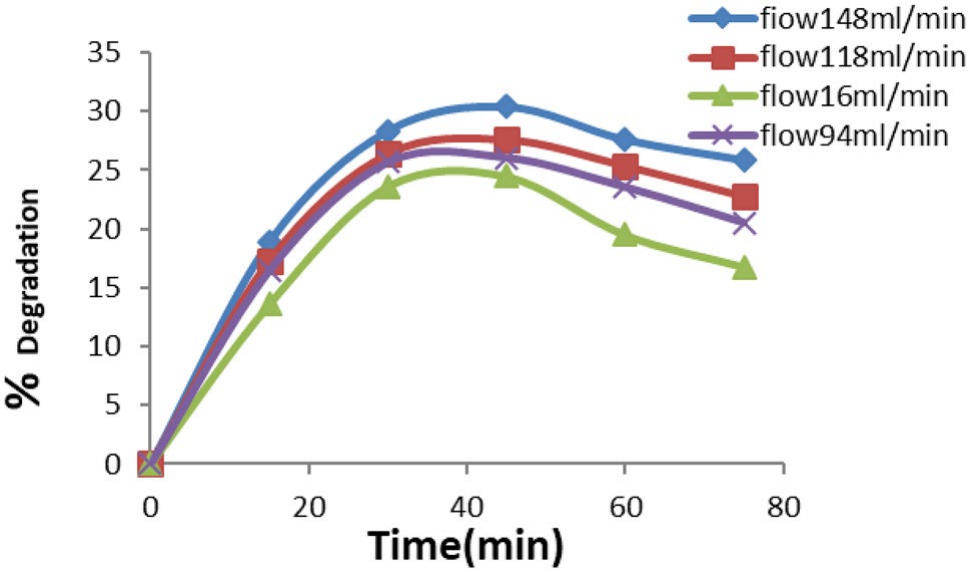
Effect of flow rate on the photocatalytic degradation of the dye

The oxidation of the Eosin Yellow dye in the tubular photocatalytic reactor was thoroughly examined. Experiments were conducted without catalyst, Eosin Yellow dye concentration was fixed at 100 ppm and four different flowrates were selected (148, 118, 94, and 16 mL min^−1^ at pH = 8.5 as shown in Fig. 3.

Examination of Fig. 3 shows that higher percentage degradation is obtained at higher flow rate. A maximum of 28.86% degradation is obtained for flow rate 148 ml/min.

#### Effect of pH Value

**Fig. 4:**
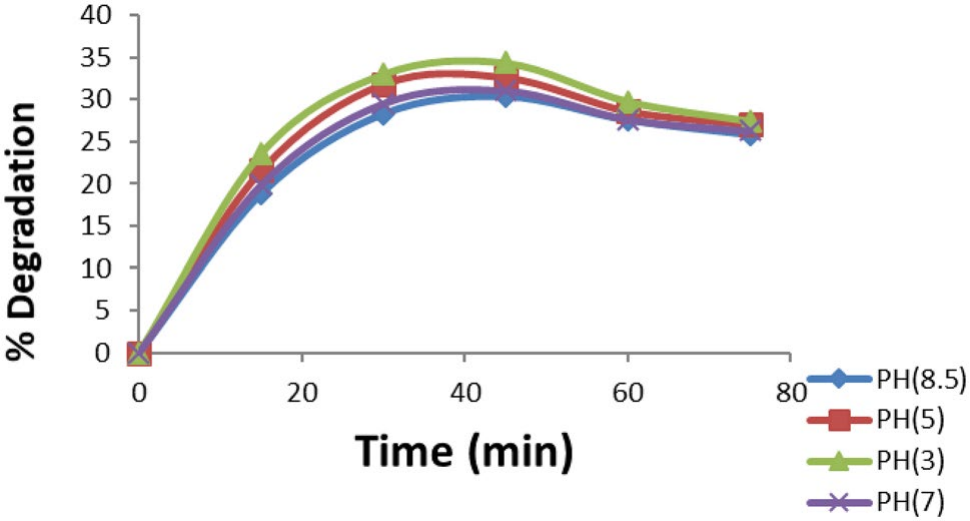
Effect of pH value on % degradation of 100 ppm dye solution

A set of experiments was performed at a flow rate of 148 mL/ min with different values of pH (3, 5, 7, and 8.5). The results of this test are shown in Fig. 4. It has been postulated that the dye degradation is affected by the value of pH and dye degradation was better in the acidic medium than in alkaline medium. This agrees with the work of Viswanathan B [23].

### Photocatalytic Efficiency Studies of EY Dye Using ZnO NPs

Degradation of an aqueous solution of EY dye in presence of ZnO nanoparticles irradiated with UV light at different time intervals was followed. Several reaction variables including initial dye concentration, flow rates, catalyst dose and pH value were studied.

#### Effect of Initial EY Concentration

**Fig. 5:**
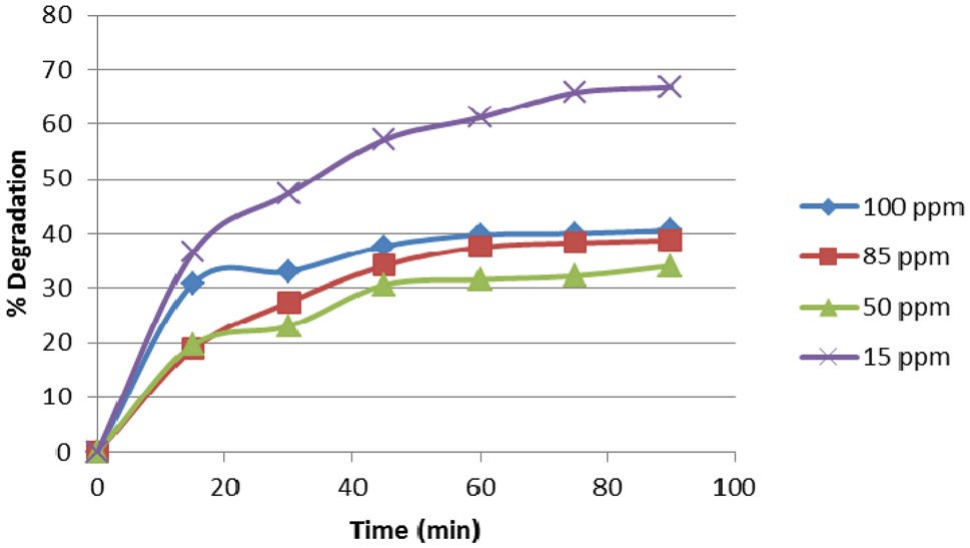
Effect of initial concentration of EY dye on the degradation efficiency

Experiments were run using various concentrations of dye (15–85 ppm) at catalyst dose 0.4 g/L ZnO NPs, pH value of 3, flow rate 148 mL/min and 90 min reaction time. Results are shown in Fig. 5 and the results show that above 15 ppm dye concentration the % degradation decreased. The possible cause was ascribed to the dye solution's reduced transparency at high concentrations, which may have led to the dye molecules to absorb UV light and making them less accessible for OH-mediated photocatalytic degradation. It is clearly shown in Fig. 5 that the dye oxidation is higher at the early stage of contact time followed by a decrease in the reaction rate. This conclusion is consistent with the findings of Abdellah et al. [24] who concluded that the initial concentration of EY dye affects the degradation efficiency.

This is justified by the fact that as the initial dye concentration rises, so does the number of adsorbed dye molecules on the surface of ZnO NPs. As a result, dye molecules occupy the active sites of the catalyst, lowering degradation efficiency since the activity of the active surface of the ZnO NPs decreases. This is attributed to a decrease of hydroxyl radicals, which is the major cause of the reaction, after the initial period of reaction.

Another possible reason is the screening effect of the UV light, which increases with the increase in the intensity of the color (higher dye concentration). The screening of UV light results from the absorption of a fraction of UV light by the chromophores of the dye molecules. Therefore, less photon’s energy reaches the active sites on the catalyst, which results in a lower number of OH produced. Moreover, intermediate chemicals produced during the degradation process rise as the dye concentration rises, and they may consume some of the active radicals that are meant to react with the dye molecules. So the total efficiency of decolorization falls.

#### Effect of Photocatalyst Concentration

Under the preferred conditions of pH (3), dye concentration 50 ppm, and flow rate 148 mL/min, the impact of different catalyst concentrations (0.2–0.8 mg/L) on degradation efficiency was assessed.

**Fig. 6:**
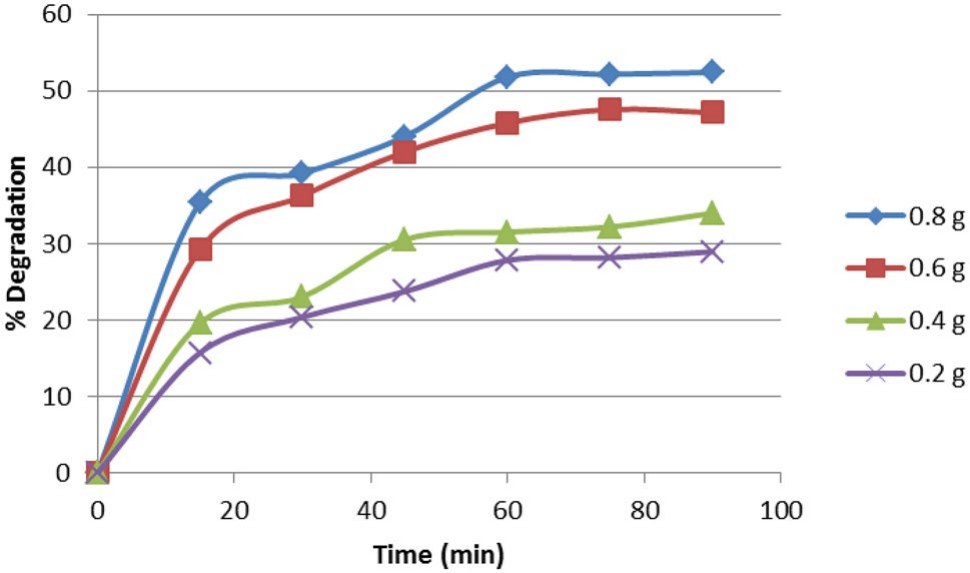
Effect of ZnO dosage on the photodegradation process efficiency

According to Fig. 6, the less catalyst amount employed in the degradation process resulted in lower degradation efficiency. Consequently, as the catalyst dose was raised, degradation efficiency improved (to 52.4% at 0.8 ppm) owing to the availability of efficient and sufficient active sites, resulting in high adsorption and enhanced OH radical generation. The surface area of photocatalyst has a significant impact on its catalytic performance. As a result, finding the best dosage of catalyst is vital for improving degradation efficiency.

#### Effect of Flow Rate

**Fig. 7:**
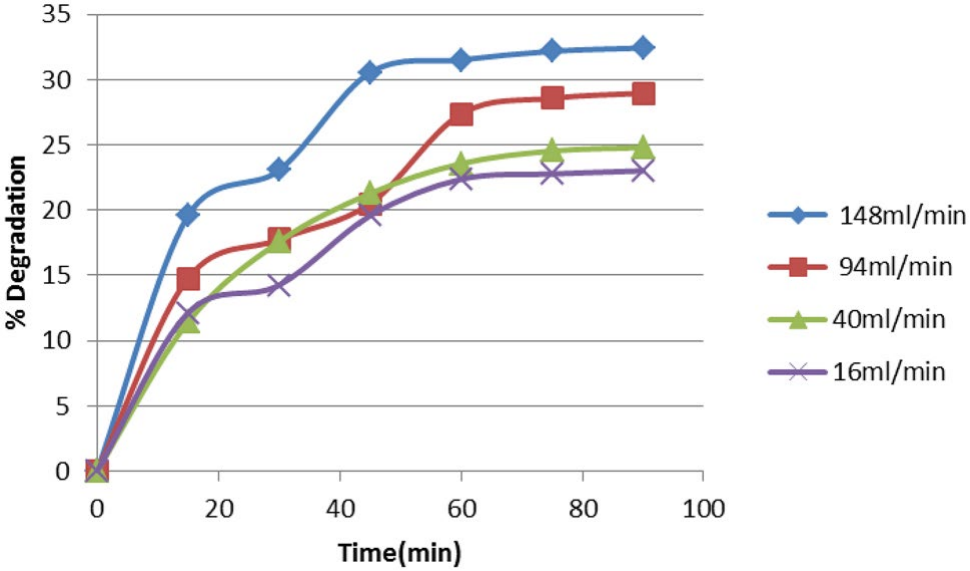
Effect of flow rate on the photodegradation efficiency of dye

A number of tests was carried out at various flow rates ranging from 16 to 148 mL/min, even while operating at the same EY dye concentration (50 mg/L) and applying the same catalyst dosage (0.4 g/L). The results of this test are shown in Fig. 7. Examination of Fig. 7 clarifies that the percent removal of the dye rises by raising the flow rate. A percentage degradation of 32.4% is reached at flow rate 148 mL/min.

This could be due to the fact that the exposure time to UV radiation is proportional to the flow rate and consequently to the OH radicals produced. Thus, the dye removal increases when more OH radicals are attained, i.e., with higher flow rates.

#### Effect of pH Value

**Fig. 8:**
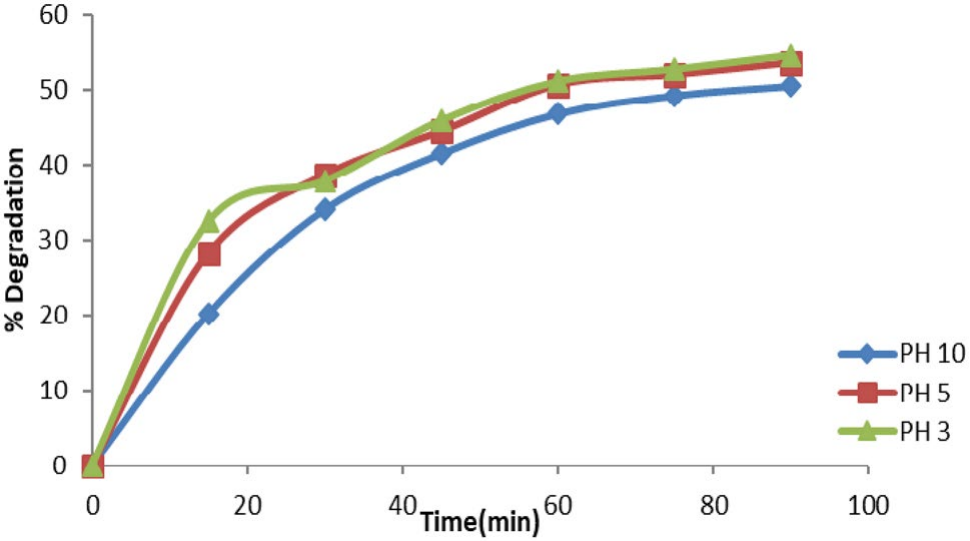
Percentage dye removal for different solution pH values

The pH of the dye solution is changed depending on the nature of the dye (cationic, anionic, or neutral) to improve the dyeing process and guarantee a strong adherence of dye molecules to the material to be colored. The pH influence on the photocatalytic activity of ZnO Nanoparticles was examined, with the findings given in Fig. 8. It can be observed that a pH value of 3 was identified to be the best. When the pH value was increased from 3 to 10, the decolorization effectiveness of EY dropped from 54.5% to 50.5%. Based on the point of zero charge of ZnO, the pH influence on the photocatalytic activity of the ZnO photocatalyst may be addressed. pH changes cause the redox potentials of the valence and conduction bands to fluctuate, potentially affecting interfacial charge transfer. The surface of ZnO photocatalyst is positively charged at low pH, but becomes negatively charged at high pH. This agrees with the work of Kazeminezhad et al. [25].

#### Effect of Presence of Catalyst

The results of two similar tests, varying only in the presence of catalyst in one of them, are compared to investigate the effect of presence of photocatalyst. Thus, in one test the dye solution is only UV illuminated while in the other test the reaction is UV-ZnO NPs catalyzed.

Dye concentration was fixed at 100 ppm, pH at 3 and flow rate of 148 mL min^−1^ was selected. In one experiment, catalyst of concentration 0.8 g was added.

**Fig. 9:**
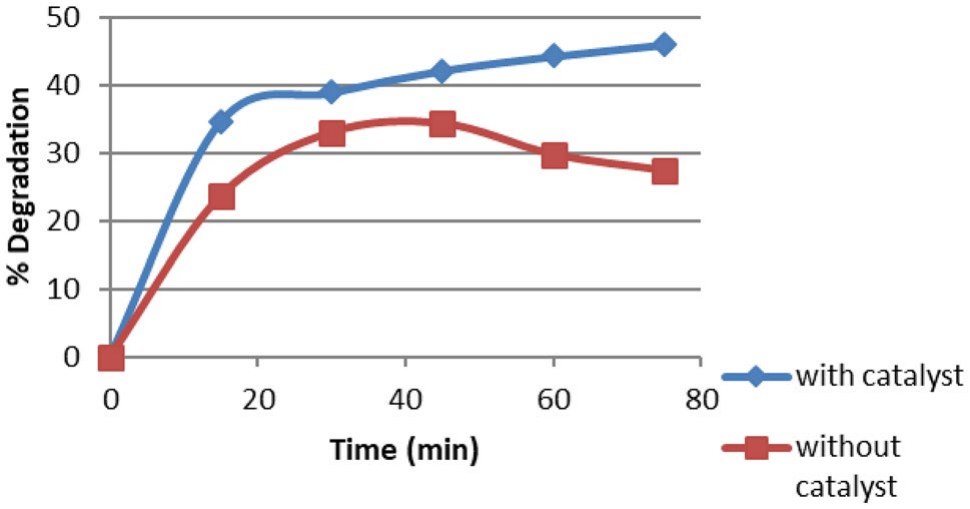
Comparison of UV- illumination and UV-ZnO NPs catalyzed dye degradation

The results of this comparison test are given in Fig. 9. Examination of this figure indicates that UV oxidation resulted in 27.43% dye degradation, compared to 45.95% for UV- ZnO NPs catalyzed process. This agrees with the work of Cambrussi et al. [26].

### Statistical Optimization of the Operating Parameters

#### The Response Surface Model of the % Degradation Outcome

The experimental values for the process response (% Degradation) at the design points are given in Table 2.

**Table 2:** Experimental design for dye removal process (constant time = 90 min)

Run	Amount of catalyst (g)	Dye concentration (ppm)	Flow rate (ml/min)	% Degradation
1	0.4	85	40	31.64
2	0.4	85	148	38.67
3	0.2	50	40	24.8
4	0.6	15	94	71.42
5	0.4	50	94	43.96
6	0.2	85	94	23.99
7	0.6	85	94	41.91
8	0.2	15	94	47.99
9	0.6	50	40	44.75
10	0.6	50	148	47.13
11	0.4	15	40	51.39
12	0.4	15	148	66.82
13	0.4	50	94	44.22
14	0.4	50	94	46.22
15	0.2	50	148	28.95

A Box–Behnken design shown in Table 2 lets the exploration of the second-order model expressed in a mathematical polynomial equation (Eq. 1) where the response variable (% Degradation) is evaluated as a second-order function of ZnO NP dosage (A), initial dye concentration (B) and flow rate (C) and assessed as the sum of a constant, three coefficients of first-order (terms in A, B and C), three coefficients of interaction (AB), (AC) and (BC) and the three coefficients of the second order (AA, BB and CC) as illustrated in Eq. (1).

1 $$\begin{aligned}\% \;Degradation=&+\; 44.80+9.93A-12.68B+3.62C\\ &-1.38AB-0.44AC-2.10BC\\ &-{4.60A}^{2}+{6.13B}^{2}-{3.80C}^{2}\end{aligned}$$

**Fig. 10:**
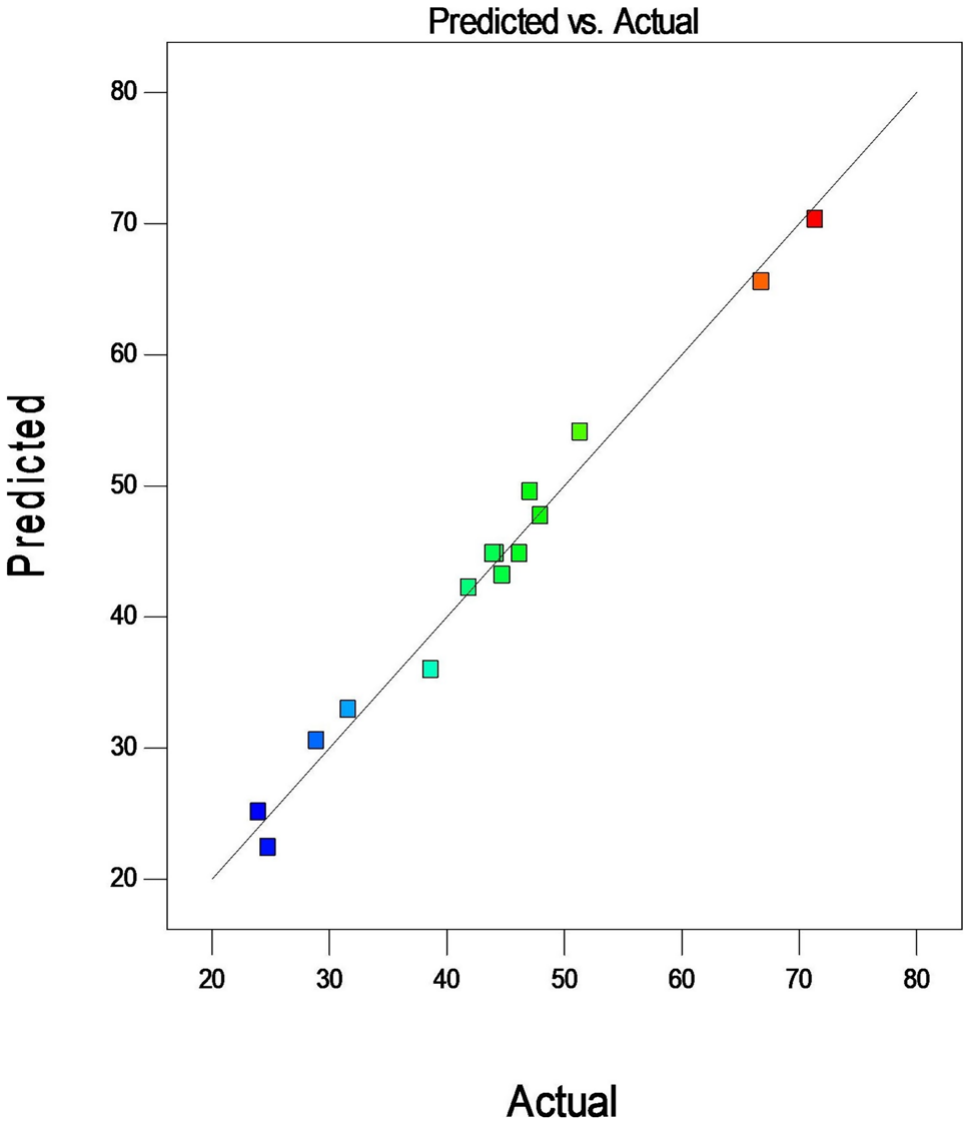
Predicted and actual values for the developed model

Figure 10 shows that the predicted values for the % Degradation response are reasonably close to the actual values, thus, confirming the consistency of the models established for creating a relationship concerning the independent process variables (Amount of catalyst, Dye concentration and Flow rate) and the response (Percent dye removal).

The statistical significance of the % degradation model was assessed by ANOVA (Table 3). Table 3 indicates that the Model F-value of 34.67 implies that the model is significant. There is only a 0.06% chance that an F-value this large could occur due to noise. Values of "Prob > F" less than 0.050 indicate that model terms are significant. In this case A, B, C, A^2^, B^2^, C^2^ are significant model terms.

**Table 3:** ANOVA for Response Surface Quadratic Model

Source	Df	SS	MS	F-value	P-value
					prob > F
Model	9	2497.05	277.45	34.67	0.0006
Residual	5	40.01	8		
Total	14	2735.05			
		0.984			

*Df* Degree of freedom, *SS* Sum of squares, *MS* Mean Squares

#### Effect of Process Parameters

**Fig. 11:**
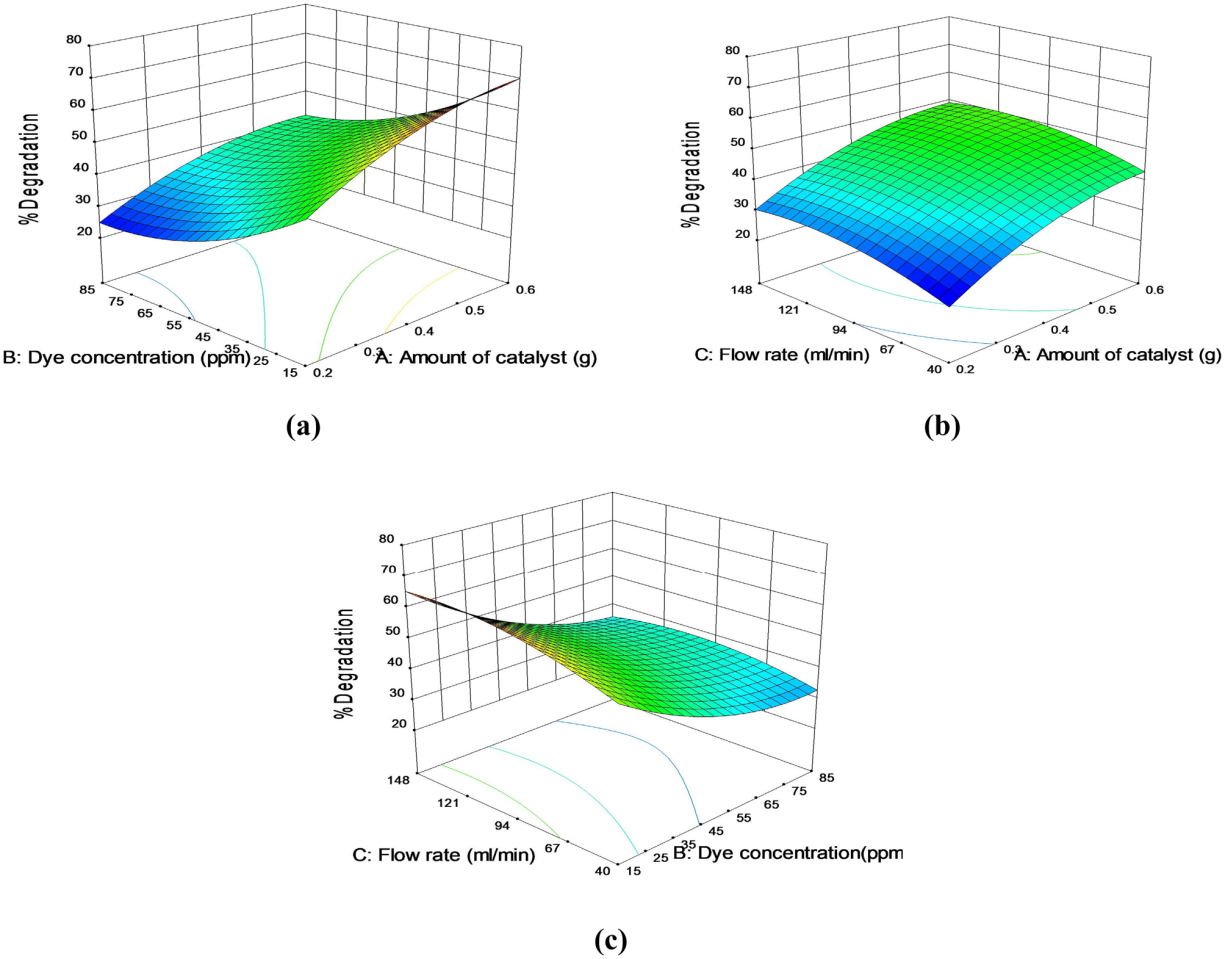
The response surface plot for the effect of amount of catalyst and dye concentration (**a**), amount of catalyst and flow rate (**b**), dye concentration and flow rate (**c**) on % Degradation

The response surface curves for the predicted model were drawn to show the effects of the independent variables (Amount of catalyst, Dye concentration and Flow rate) on the dependent variables (% Degradation). These 3D surface graphs (Fig. 11(a, b, c)) show the effect of two variables within their studied ranges, with the other variable fixed to the zero level.

Figure 11 indicates that there was a relative significant interaction between every two variables (amount of catalyst and dye concentration, amount of catalyst and flow rate, dye concentration and flow rate). The % degradation generally increased as the amount of catalyst increased. Also, the same result is observed for the flow rate; the % degradation increased with increasing the flow rate. While, the % degradation decreased as the dye concentration increased.

When the ZnO dose was increased, the maximum removal rate was increased, as shown in Fig. 11(a). Higher dye concentrations, on the other hand, had a detrimental impact on removal efficiency. So, an increase in dye concentrations has a significant influence on the dye removal rate efficiency. As a result, the appropriate dose of ZnO NP and dye concentrations for each procedure should be determined. The results in Fig. 11 demonstrate a favorable improvement in the removal rate due to the interaction between the ZnO NP dose and the aqueous solution flow rate.

Figure 11(c) shows that a decrease in the dye concentration value and an increase in the flow rate improve the dye removal efficiency. Thus, ZnO NP dosage and dye concentration together with the flow rate of the solution should be in optimal values to maximize the dye removal rate.

#### Optimization of the Operating Parameters to Enhance Dye Removal Process

The optimum values were identified to achieve the maximization of the % degradation based on the relevant developed model.

The optimum values were as follows: 0.59 g, 15.75 ppm and 136.12 ml/min for amount of catalyst, dye concentration and flow rate, respectively. The maximum percent degradation achieved at these conditions was 71.44%.

## Conclusion

The following conclusions could be made from the present worrk.

1. Higher percentage degradation is obtained at higher flow rate. A maximum of 28.86% degradation is obtained for flow rate 148 ml/min.
2. The acidic medium gives higher percentage degradation than alkaline medium.
3. Dye solutions of more than 15 ppm concentration show lower percentage degradation.
4. As the catalyst dose was raised, degradation efficiency improved.
5. The percentage removal of the dye rises by raising the flow rate.
6. The percentage of degradation is enhanced when using UV- ZnO NPs catalyzed process than when using UV oxidation only.
7. Behnken-Design under category of RSM is applied and then RSM is used to optimize the operating parameters affecting the degradation of Eosin Y dye.
8. The optimum values were as follows: 0.59 g, 15.75 ppm and 136.12 ml/min for amount of catalyst, dye concentration and flow rate, respectively. The maximum percentage degradation achieved at these conditions was 71.44%.

## Data Availability

N/A.
